# Metabolism Regimes in Regulated Rivers of the Illinois River Basin, USA

**DOI:** 10.1038/s41597-024-03037-1

**Published:** 2024-02-15

**Authors:** Judson W. Harvey, Jay Choi, Katherine Quion

**Affiliations:** grid.2865.9 0000000121546924 U.S. Geological Survey, Earth System Processes Division, Reston, VA USA

**Keywords:** Freshwater ecology, Water resources

## Abstract

Metabolism estimates organic carbon accumulation by primary productivity and removal by respiration. In rivers it is relevant to assessing trophic status and threats to river health such as hypoxia as well as greenhouse gas fluxes. We estimated metabolism in 17 rivers of the Illinois River basin (IRB) for a total of 15,176 days, or an average of 2.5 years per site. Daily estimates of gross primary productivity (GPP), ecosystem respiration (ER), net ecosystem productivity (NEP), and the air-water gas exchange rate constant (K_600_) are reported, along with ancillary data such as river temperature and saturated dissolved oxygen concentration, barometric pressure, and river depth and discharge. Workflows for metabolism estimation and quality assurance are described including a new method for estimating river depth. IRB rivers are dominantly heterotrophic; however, autotrophy was common in river locations coinciding with reported harmful algal blooms (HABs) events. Metabolism of these regulated Midwestern U.S. rivers can help assess the causes and consequences of excessive algal blooms in rivers and their role in river ecological health.

## Background & Summary

Aquatic metabolism measures the balance between organic carbon accumulation by primary productivity of algae and other autotrophs and the rate of carbon removal by respiration of autotrophs and heterotrophs such as bacteria. River metabolism is relevant to assessing causes and consequences of eutrophication such as hypoxia, serving as an early warning indicator of changing river functions and health as well as indicating shifts in greenhouse gas emissions^1,2^. Here we focused on metabolism of regulated rivers in the Illinois River basin (IRB) where river algal blooms and associated toxins have been reported^3–7^. To quantify metabolism, the rate of oxygen production and consumption in the aquatic system is measured over time to estimate gross primary productivity (GPP) and ecosystem respiration (ER). GPP is a positive quantity that estimates the daily growth rate of autotrophs and ER is a negative quantity that estimates the daily rate of organic carbon loss by organism respiration including respiration of autotrophs and respiration associated with microbial decomposition of detrital organic matter. The sum of GPP and ER is the net ecosystem productivity (NEP), which estimates the daily balance between organic carbon build up and depletion in the system by primary productivity and respiration. To use the oxygen balance method to estimate metabolism it is necessary to also quantify the rate of dissolved oxygen exchange with the atmosphere, which depends on water temperature and atmospheric pressure as well as water mixing and turbulence. As methods improve to measure metabolism, the numbers of studies have substantially increased. However, most long-term estimates in flowing waters are confined to small streams and wadable rivers^2^.

**Fig. 1:**
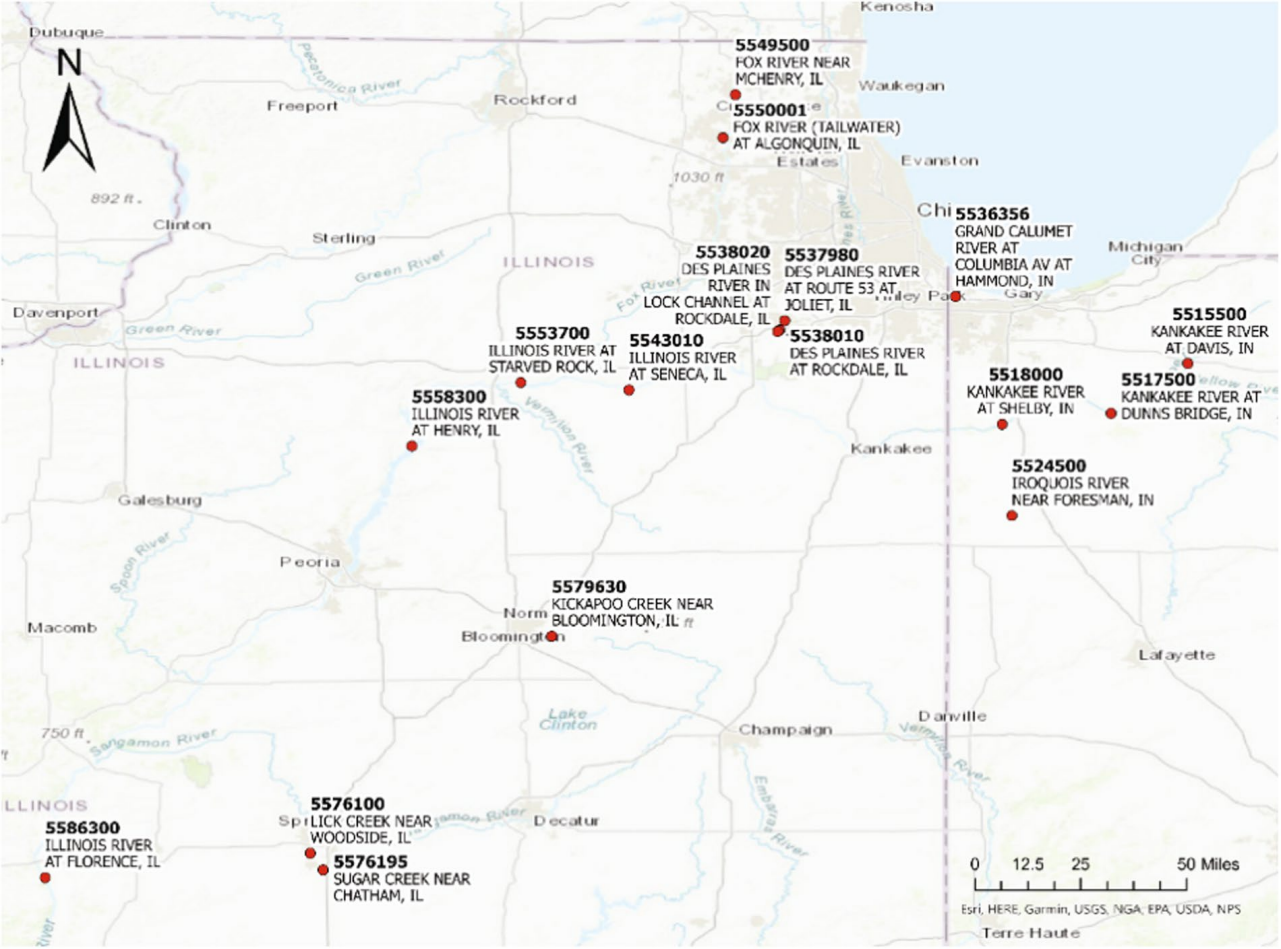
Seventeen river sites in the Illinois River Basin (IRB) selected for metabolism modeling. Site names and numbers reference data sourced from the U.S. Geological Survey National Water Information System (USGS | National Water Dashboard).

For the present study we estimated aquatic metabolism at 17 river sites in the Illinois River basin (IRB)^8^ that encompassed extensive agricultural areas and a major metropolitan area in northeastern Illinois as well as agricultural and suburban areas in northwestern Indiana and in southern Wisconsin that drain to the Illinois River (Fig. 1, Table 1).

**Table 1:** Site name, U.S. Geological Survey National Water Information System (NWIS) site number, geographic coordinates, presence of lock and dam regulation, and period of data availability for metabolism modelling at the study of 17 IRB river sites.

Site Name	USGS NWIS Number	Latitude	Longitude	Presence of Lock and Dam (LD) Regulation	Period of Data Availability
**ILLINOIS RIVER** AT FLORENCE, IL	05586300	39.63278	−90.60778	downstream of LaGrange LD	2012-06-02 2021-01-01
**ILLINOIS RIVER** AT HENRY, IL	05558300	41.10722	−89.35611	between Marseilles and Peoria LD	2018-06-06 2020-12-31
**ILLINOIS RIVER** AT STARVED ROCK, IL	05553700	41.32476	−88.98397	between Marseilles and Peoria LD	2018-06-05 2020-12-31
**ILLINOIS RIVER** AT SENECA, IL	05543010	41.29972	−88.61417	between Dresden and Marseilles LD	2013-06-27 2020-12-13
**FOX RIVER** NEAR MCHENRY, IL	05549500	42.31002	−88.25147		2018-08-27 2020-10-22
**FOX RIVER** (TAILWATER) AT ALGONQUIN, IL	05550001	42.16194	−88.29389		2016-06-30 2018-10-11
**DES PLAINES RIVER** AT ROUTE 53 AT JOLIET, IL	05537980	41.53639	−88.08250	between Chicago CAWS and Brandon LD	2017-11-16 2020-12-31
**DES PLAINES RIVER** AT ROCKDALE, IL	05538010	41.50500	−88.09972	between Chicago CAWS and Brandon LD	2015-08-14 2017-03-21
**DES PLAINES RIVER** IN LOCK CHANNEL AT ROCKDALE, IL	05538020	41.50000	−88.10694	between Chicago CAWS and Brandon LD	2015-08-14 2020-12-31
**KANKAKEE RIVER** AT SHELBY, IN	05518000	41.18281	−87.34031		2015-12-04 2020-12-31
**KANKAKEE RIVER** AT DUNNS BRIDGE, IN	05517500	41.22004	−86.96836		2016-04-08 2020-12-31
**KANKAKEE RIVER** AT DAVIS, IN	05515500	41.38964	−86.70617		2013-12-04 2020-12-31
**IROQUOIS RIVER** NEAR FORESMAN, IN	05524500	40.87059	−87.30669		2018-12-14 2020-12-31
**GRAND CALUMET RIVER** AT COLUMBIA AV AT HAMMOND, IN	05536356	41.61861	−87.49983		2020-03-18 2020-10-12
**LICK CREEK** NEAR WOODSIDE, IL	05576100	39.71554	−89.70244		2015-06-29 2018-11-29
**SUGAR CREEK** NEAR CHATHAM, IL	05576195	39.65908	−89.65894		2015-06-13 2018-11-29
**KICKAPOO CREEK** NEAR BLOOMINGTON, IL	05579630	40.45833	−88.8775		2011-03-24 2015-07-14

The selected IRB sites represent a variety of river sizes and characteristics, including mainstem sites on the Illinois River as well as several large tributaries and a few smaller streams. The Illinois River is substantially regulated by a series of locks and dams to maintain minimum water levels for navigation through the upper Illinois River as it enters the Des Plaines River tributary and headwaters of the Chicago Area Waterway System (CAWS). Not surprisingly, water quality and ecological conditions are substantially impaired in IRB rivers, including high nutrients and suspended sediments^3,4^. Large tributaries of the Illinois River include the Kankakee River which drains large areas of corn and soybean agriculture and has been dredged and straightened to increase its conveyance, and now has significant problems with high turbidity and sedimentation^3^. The Fox River flows through agricultural areas in southern Wisconsin and then traverses the western edge of the Chicago urban corridor before joining the Illinois River^5^. Dam storage in the Illinois and Fox Rivers maintains significant water depths and lengthens water residence times while also increasing water clarity^4^. Recently, excessive plankton blooms and associated algal toxins have been observed in the Illinois and Fox Rivers^5–7^.

The type of autotrophs in water bodies (e.g., benthic vs. planktonic algae vs. submerged aquatic vegetation) depends on light availability which is affected by tree and bank shading and water-column light attenuation, disturbance frequency and severity, and other factors^1,2^. Benthic algae are usually thought to dominate GPP in streams and small rivers where the river bed is illuminated^1,2^. Many benthic algal species are adapted to shading by forest canopies, as well as the high-flow events that scour stream beds and disrupt GPP^2^. Planktonic algae are usually thought to dominate in lakes, reservoirs, and estuaries; however, the expectation for large rivers is less clear^9^. However, unshaded rivers with low or moderate turbidity have the potential for high water-column GPP from phytoplankton growth^8,9^.

Phytoplankton and harmful algal blooms (HABs) have increasingly been observed in large rivers and reservoirs of the Midwest and Great Plains areas of the United States such as the Kansas, Ohio, and Mississippi Rivers^10–13^, as well as in the Illinois River^5–7^ and elsewhere^14,15^. Flow extremes are moderated in regulated rivers such as the Ohio, Mississippi, and Illinois Rivers where locks and dams lengthen the water residence time and increase the water clarity in the quiescent river pools between the dams^16,17^. Regulated rivers also often have abundant nutrient supply^3–6^ which can support phytoplankton blooms during low flow periods, when water residence time is prolonged, when water is warmer than average, and when turbidity from suspended sediments is often at its lowest^16,17^.

Chlorophyll-*a* (chl-*a*) is often used as a measure of phytoplankton, however, riverine chl-*a* can reflect a myriad of algal types and is not distinctly diagnostic of phytoplankton^18^. Also, the relationship between chl-*a* and autotrophic biomass may vary greatly depending on light, nutrients, temperature, and other factors^19^. Use of metabolism metrics in rivers can improve understanding of the drivers of river algal blooms^20^ and can help anticipate future changes in river health^21–23^. For example, changes in the sign of NEP and in the temporal correlation of GPP and ER can signal changes in the relative importance of phytoplankton versus submerged aquatic vegetation as dominant primary producers in rivers^21^.

**Fig. 2:**
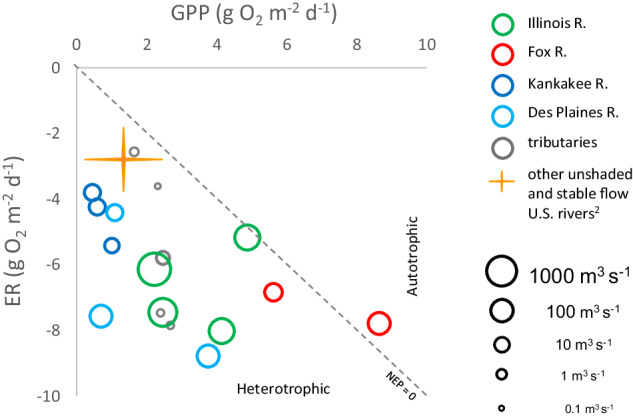
Average gross primary productivity (GPP) versus ecosystem respiration (ER) in regulated rivers and various tributaries of the Illinois River Basin (IRB), USA. IRB study rivers are distinguished by symbol color with symbol size scaled by mean river discharge. Dashed line denotes where net ecosystem productivity (NEP) equals zero and separates heterotrophic from autotrophic conditions. The orange cross shows the approximate inter-quartile range of average GPP and ER for 18 “unshaded and stable flow” rivers in the United States^2^.

Most previous metabolism estimation in rivers was focused on streams and small rivers^2^. To motivate further use of the IRB metabolism data^8^, we plotted long-term average metabolism for 17 IRB river sites (Fig. 2). Like many heterotrophic streams and rivers that process substantial inputs of allochthonous organic matter^1,2,9,23^, the metabolism of IRB rivers was generally heterotrophic (Fig. 2).

The overall productivity of IRB rivers (mean GPP = 2.77 g O_2_ m^−2^ d^−1^) was representative of the relatively high productivity of a subgroup of 18 high productivity “unshaded and stable flow” rivers evaluated as part of a study of 220 rivers and streams^2^ (Fig. 2). Productivity was generally higher in unshaded and stable flow rivers compared to most other streams and rivers because of greater light availability and because smaller variations of river discharge disturb autotrophs less frequently^2^. Only one of our IRB study rivers (Fox R. with an average GPP of 7.13 g O_2_ m^−2^ d^−1^) was a standout in productivity compared to the unshaded and stable flow subgroup. However, nearly all IRB rivers were substantially higher (more negative) in ER (mean ER = −6.05 g O_2_ m^−2^ d^−1^) compared with the unshaded and stable flow subgroup from the broader analysis^2^ (Fig. 2).

Our dataset indicates that IRB river metabolism is heterotrophic overall (mean IRB river NEP = −3.28 g O_2_ m^−2^ d^−1^), however, IRB rivers were intermittently autotrophic, accounting for between 1 and 56% of the measured days (Table 6 and Fig. 2). At one extreme the Kankakee and Des Plaines Rivers were usually strongly heterotrophic and were only autotrophic on 1% and 5% of days, respectively. At the other extreme the Illinois River and Fox Rivers were autotrophic 33% and 43% of days, respectively. Tributaries were intermediate in their autotrophy ranging between 12% and 23% of days (Table 6 and Fig. 2).

Frequent autotrophy in rivers is an indicator but does not in itself imply phytoplankton production^21^. However, the correspondingly high chlorophyll-*a* (chl-*a*) measurements in the Illinois and Fox Rivers^6^ compounded with visual reporting and analytical determinations of planktonic algae^5,7^ indicate that phytoplankton blooms are common in the IRB. We encourage further analysis of our IRB river metabolism data set^8^ in the context of water quality^24,25^ and river conditions^26–28^ to better understand the triggers and consequences of riverine planktonic algal blooms, in the IRB and elsewhere.

## Methods

Initial site selection for metabolism estimation in IRB rivers was based on the availability of dissolved oxygen data accessed from the U.S. Geological Survey National Water Information System^25^ (USGS NWIS). We used the USGS | National Water Dashboard link to help identify NWIS site numbers with the needed input data. USGS scalable maps of water-quality data collection sites that are available at that site were consulted. Potential river sites were identified by searching all “stream type” sites including “streams”, “canals”, and “ditches” with at least a year of continuous collection of dissolved oxygen data (i.e., generally 15-minute intervals). Sites were excluded that were obviously not lotic in character, e.g., wetlands, ponds, gravel pits, which resulted in identifying seventeen IRB river sites that were appropriate for modeling long-term metabolism. Selected sites were linked to the National Hydrography Dataset (NHDPlus)^26^ to take advantage of documented river and catchment attributes.

We used USGS data retrieval software (dataRetrieval)^29^ to download between one and nine years of data from 17 selected IRB river sites (Table 1) including all continuous (sub-daily) measurements of dissolved oxygen concentration, water temperature, specific conductivity, continuous daily water discharge and gage height (Table 2), as well as downloading infrequently collected channel field measurements (Table 3). Barometric pressure was obtained separately through a request to NOAA^30^ using site latitude and longitude to select the closest nearby measurement location for each river site. All of the dissolved oxygen (DO) data used in this study were quality assured and approved by the USGS. The DO data are expected to be of high quality because they were collected after 2010, after the use of optical DO sensors had become standard practice. Although it did not apply to our IRB data, recently collected USGS data that is available for download is sometimes provisional and not yet quality assured.

**Table 2:** List of data sources for metabolism modeling including USGS data obtained using USGS data retrieval software^29^ and NOAA National Centers for Environmental Information, U.S. Local Climatological Data (LCD)^30^.

Parameter	Source	USGS Parameter Code	Definition	Use in stream metabolism modeling
Dissolved Oxygen	USGS NWIS	00300	Dissolved oxygen, water, unfiltered, milligrams per liter	• Estimate GPP and ER
Specific Conductance	USGS NWIS	00095	Specific conductance, water, unfiltered, microsiemens per centimeter at 25 degrees Celsius	• Convert to salinity, then used in calculation of dissolved oxygen saturation
Water Temperature	USGS NWIS	00010	Temperature, water, degrees Celsius	• Used in calculation of dissolved oxygen saturation
Discharge	USGS NWIS	00060	Discharge, cubic feet per second	• Constraining K_600_ estimates
Gage Height	USGS NWIS	00065	Gage height, feet	• Estimating river depth
Barometric Pressure	NOAA	Not applicable	Air pressure, millibar	• Used in addition to specific conductance and temperature to calculate dissolved oxygen saturation

**Table 3:** Parameters calculated from source data for metabolism modeling.

Parameter	Units	Calculation description and package::function(s) used	Required Inputs
Oxygen Saturation	percent (%)	streamMetabolizer::calc_DO_sat()	water temperature, air pressure (from NOAA), salinity
Light Intensity	photon density (μmol m^−2^ s^−1^)	streamMetabolizer::calc_light()	solar time, latitude, longitude
Solar Time	Mean solar (exactly 24 hours between solar noons)	streamMetabolizer::convert_UTC_to solartime()	time in Coordinated Universal Time (UTC), longitude
River Depth	meters	Develop linear rating curves to estimate river depth and velocity from channel field measurements obtained using dataRetrieval::readNWISmeas())	Required to use dataRetrieval::readNWISmeas():
			1. USGS site number
			2. start date
			3. end date
		or	
		Use the equation (*h*_*hgc*_ = c*(Q)^*f*^) where *h*_*hgc*_ is the river depth estimated by hydraulic geometry, *c* and *f* are hydraulic geometry coefficients, and Q is continuous discharge	Use dataRetrieval::readNWISmeas() to download field measurements
			1. channel width
			2. channel cross sectional area
			3. discharge
			4. gage height

To model metabolism we took advantage of recent advancements with state-space models that simultaneously estimate three unknown metabolism variables, GPP, ER, and K_600_^31–33^. Generally, models converge better and produce physically realistic estimates when GPP > rate of air-water oxygen exchange, a condition that accentuates diel variation in dissolved oxygen concentration and increases the signal-to-noise ratio that aids model identification of the competing influences of GPP, ER, and K_600_. Nevertheless, metabolism estimation remains a challenge because of the potential difficulties in estimating three co-related parameters from a single oxygen time series.

To model metabolism in IRB rivers we used the *streamMetabolizer* R package (https://github.com/USGS-R/streamMetabolizer), a widely tested and well documented state-space metabolism model^33^. This model uses the one-station modeling approach that assumes that sensor data collected at a single point in a river is representative of a well-mixed water column. The accuracy of DO measurements is also important; however, the measurement accuracy has improved substantially since high-quality optical dissolved oxygen sensors began being used routinely (approximately 2005). Furthermore, the model does not quantify anaerobic respiration that is sometimes significant in low-oxygen rivers. In addition to assuming well-mixed conditions, the one-station modeling approach assumes homogenous upstream conditions affecting metabolism for a distance that is assumed to be proportional to *v*/*K* where *v* is stream velocity and *K* is the gas exchange coefficient.

The governing mass balance equations equate the instantaneous rate of change in DO [O_2_] in the river with the sum of the rates of DO inputs and outputs by metabolism and gas exchange^32^. Expressed as volumetric rates, the mass balance for DO is:

1 $$\frac{d[{O}_{2}]}{dt}={P}_{t}+{R}_{t}+{D}_{t}$$

where d[O_2_]/dt is the rate of change in water column O_2_ [mg O_2_ L^−1^ d^−1^]; P_t_ is the instantaneous volumetric rate of oxygen addition by gross primary production [mg O_2_ L^−1^ d^−1^]; R_t_ is the instantaneous volumetric rate of oxygen removal by respiration [mg O_2_ L^−1^ d^−1^]; and D_t_ is the instantaneous volumetric rate of air-water oxygen exchange [mg O_2_ L^−1^ d^−1^]. By the definition, P_t_ should be greater than or equal to zero, R_t_ should be less than or equal to zero, and gas exchange, D_t_, can take either sign. The *streamMetabolizer* model^33^ restructured the oxygen balance expressions by using long-term oxygen times series to estimate daily metabolism variables through the solution of the following equations:

2 $${P}_{t}={\boldsymbol{GPP}}\times \frac{1}{h}\times \frac{\left({t}_{1}-{t}_{0}\right)\times PPF{D}_{t}}{{\int }_{u={t}_{0}}^{{t}_{1}}PPF{D}_{u}{d}_{u}}$$

3 $${R}_{t}={\boldsymbol{ER}}\times \frac{1}{h}$$

4 $${D}_{t}={K}_{2,t}\times \left({O}_{sat,t}-{O}_{mod,t}\right)$$

5 $${K}_{2,t}={{\boldsymbol{K}}}_{{\bf{600}}}\times {\left(\frac{{S}_{A}+{S}_{B}{T}_{t}+{S}_{C}{T}_{t}^{2}+{S}_{D}{T}_{t}^{3}}{600}\right)}^{-0.5}$$

where GPP is the daily areal average rate of primary production (g O_2_ m^−2^ d^−1^), ER is the daily areal average rate of respiration [g O_2_ m^−2^ d^−1^], and K_600_ is the daily average gas exchange rate constant normalized for molecular properties and temperature to a Schmidt number of 600 [day^−1^]. Variables with subscript *t* are instantaneous values that are typically estimated from 15-minute interval measurements. The rate of gas exchange, *D*_*t*_, is the product of the rate constant and the deficit between actual and saturated concentrations of dissolved O_2_. Rather than fit actual gas exchange, i.e., the K_2,t_ value, the model fits K_600_, so that only one standardized gas-exchange-related parameter per day need be reported that still captures and reflects the within-day variation in gas exchange rates caused by diel variation in temperature. Additional variables are *h*, mean river depth representing the width and upstream length of the reach affecting the oxygen balance [m]; PPFD, photosynthetic photon flux density [μmol photons m^−2^ d^−1^]; *O*_*sat,t*_, saturated O_2_ concentration [mg O_2_ L^−1^]; *O*_*mod,t*_, model estimated O_2_ concentration [mg O_2_ L^−1^]; *K*_2,t_, O_2_-specific and temperature specific gas exchange coefficient [day^−1^]; *T*_*t*_, water temperature [°C]; and *S*, Schmidt number coefficients: *S*_*A*_ = 1568, *S*_*B*_ = −86.04, *S*_*C*_ = 2.142, and *S*_*D*_ = −0.0216. The solution approach is described in detail in Appling *et al*.^33^.

### River depth estimation

River depth is necessary for metabolism estimation and the accuracy of depth estimation has a directly proportional effect on the estimation accuracy of GPP and ER. An approach previously underutilized for depth estimation in multi-river metabolism studies is using channel field measurements by the U.S. Geological Survey. We used a linear rating curve approach for estimating river depth that was based on USGS field measurements of channel width, channel area, gage height, channel discharge and channel cross-section average velocity. We obtained those field measurements from USGS NWIS^25^ using the dataRetrieval^29^ function “readNWISmeas()” that referenced USGS NWIS site number and start and end date, which often returned tens of field measurements for each site during the period of interest.

To use the linear rating curve approach to estimate river depth, the cross-section averaged depth was determined for days with field measurements by dividing the measured flow cross section by the wetted channel width:

6 $${h}_{fm}={A}_{fm}/{w}_{fm}$$

where *h*_*fm*_ is the field measured river depth, *A*_*fm*_ is the field measured channel cross-sectional area, and *w*_*fm*_ is the field measured wetted width of the river.

River depth for all model days was estimated from a linear estimation equation:

7 $$h=m\cdot GH+b$$

where *h* and *GH* are river depth and measured gage height, respectively, and model coefficients m and b for this equation were determined from a linear regression of the field measured river depth against measured gage height on the days of the field measurements.

Usually, we excluded USGS field measurements rated as “poor” from the regression of field measured river depth on gage height. At some sites, however, most of the field measurements, and sometimes all of them, were rated as poor. Nevertheless, if the gaging cross section was representative of upstream conditions, we usually judged that using field measurements to estimate river depth was superior to hydraulic geometry estimation of river depth no matter what the quality rating of the field measurements. The preferred water depth estimation method for each site is noted in Table 7.

We used the linear rating curve estimation approach for estimating river depth at thirteen of the seventeen IRB river sites where the river width at the sensor location was representative of upstream conditions (see details in next section). However, four of the seventeen river sites were located at relatively narrow control sections for which river depth estimates at the sensor location were not representative of upstream conditions. For those sites we used a hydraulic geometry approach^34^ to estimate cross-section average river depth, *h*, estimated from hydraulic geometry as:

8 $${h}_{hgc}=c\cdot {Q}^{f}$$

where *c* and *f* are hydraulic geometry coefficients^35^ for each of the river reach codes (*comID*^26^) associated with our IRB river sites, and *Q* is continuous discharge at the IRB river site.

### Assessing site representativeness of river conditions

The one station method for estimating metabolism depends on the measurement site representing both local and upstream conditions that affect metabolism estimates. A well-mixed water column, both vertically and laterally, is assumed with longitudinal consistency in river physical and biological conditions^34^. Those assumptions have been examined theoretically^36^ but are not often tested at field sites. For the present study we assessed the consistency of river width at the oxygen sensor site with river width upstream to evaluate whether the local measured river depth was representative of upstream conditions.

It is not unusual for USGS gaging and sensor measurement cross sections to be located at “control sections” that are narrower than average for the river reach, in which case the field measurements from the cross section may differ from the reach average. Both the average river depth and average velocity could be overestimated in a narrower than average measurement cross section. We consulted the USGS “water-year summary” for each site^25^ and we visually examined the gaging cross section and upstream conditions using publicly available aerial imagery (https://www.google.com/maps). The sensor location and gaging cross section where depth was measured by USGS field crews was determined from the description provided in the water-year summary^25^. Using the imagery, we examined the consistency of river width at the measurement site for approximately 10 kilometers upstream of the oxygen measurement site. Because the regulated rivers of the IRB were relatively consistent in width, we could estimate the river depth at most sites using the linear rating curve approach as described in the previous section.

To accurately estimate river metabolism, we also had to be concerned how close the site was to upstream flow regulation structures, e.g., locks and dams, or lakes. If close enough, those features affect dissolved oxygen concentrations in ways that disrupt the river metabolic signals being modeled at the sensor site. Proximity is usually judged by estimating the “metabolism reach length”, i.e., the distance required for substantial turnover of the dissolved oxygen in the water column by gas exchange with the atmosphere. Metabolism reach length was estimated as the river distance required for 80% turnover in river dissolved oxygen by gas exchange^34^, i.e., the distance where upstream river conditions are likely to influence metabolism calculations. For each day in each river, we estimated the metabolism reach length as:

9 $${\rm{metabolism}}\;{\rm{reach}}\;{\rm{length}}=-ln\left(1-0.8\right)\frac{v}{{K}_{{O}_{2}}}$$

where *v* is the cross-section averaged river velocity in m d^−1^, and ${K}_{{O}_{2}}$ is the air-water exchange coefficient for oxygen that was calculated from the *K*_600_ using the measured water temperature and published analysis equations and coefficients^33^. Cross-section averaged river velocity was estimated by dividing daily average discharge by the estimated cross-sectional channel area for that day:

10 $$v=Q/{A}_{fm}$$

where *A*_*fm*_ is the field measured channel cross-sectional area. *A* for each modeled day was estimated using a linear estimation equation:

11 $$A=m\cdot GH+b$$

where *GH* is gage height and m and b for this equation are model coefficients determined from a linear regression of the field measured cross-sectional channel area against measured gage height for the days of the field measurements.

To compare the estimated metabolism reach length with field conditions, we measured the distance from the metabolism sensor site to the nearest upstream flow regulation structures, e.g., lock and dam, or lake, by visual inspection of publicly available aerial imagery (https://www.google.com/maps) where we used that product’s measurement tool to estimate the distance from the metabolism sensor site.

### Workflow for modeling IRB river metabolism

**Fig. 3:**
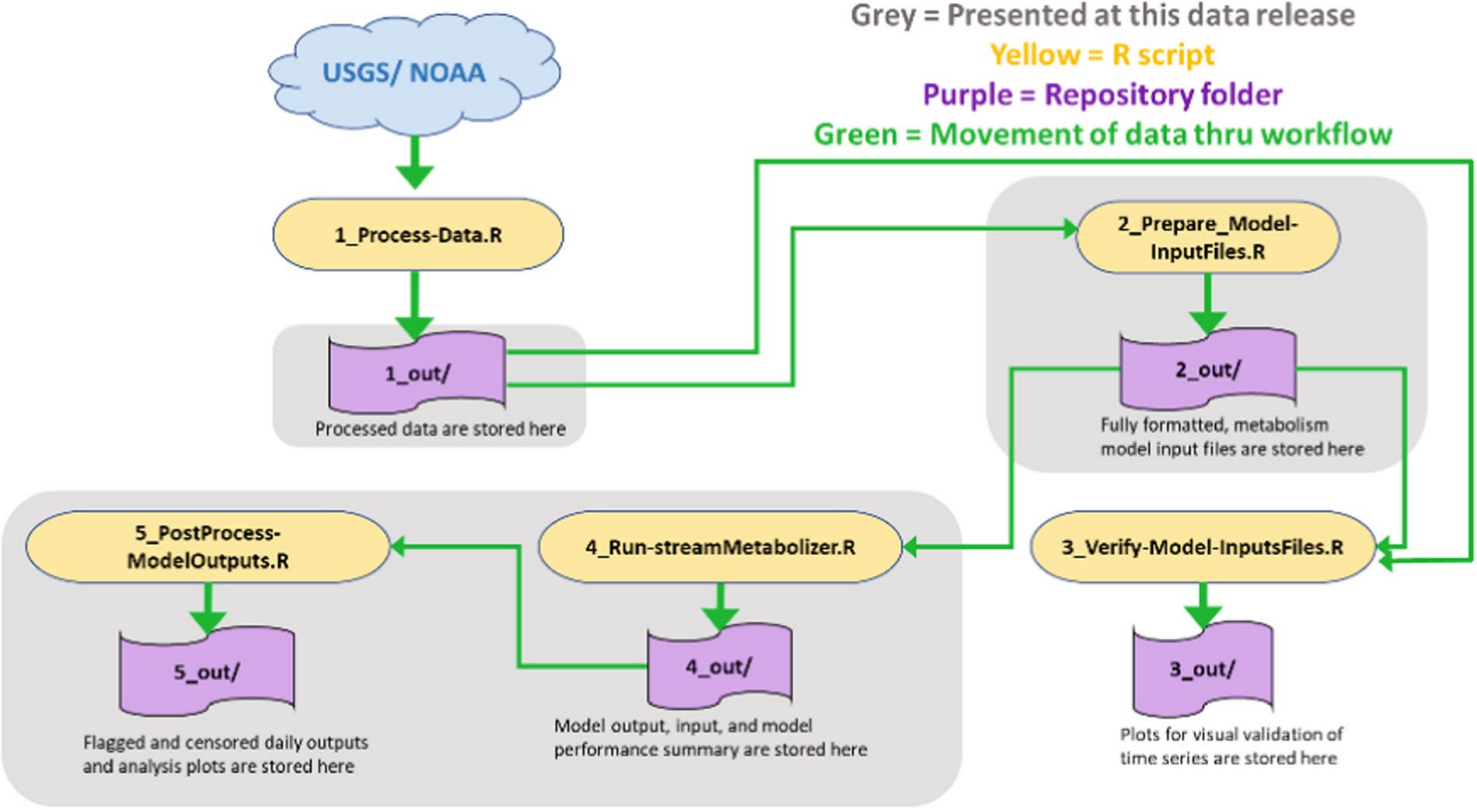
Workflow overview showing data processing and preparation of input files, model execution, post processing and quality assurance of model results.

We used R Statistical Software^37^ to process existing data to create model inputs, verify model inputs, run the *streamMetabolizer* model, and post-process and quality assure the results (Fig. 3).

The broad outlines of the workflow are documented in Fig. 3 and Table 4 and briefly summarized here. Running the first script time-matched the downloaded data, converted units, and filled time gaps less than 3 hours by linear interpolation. Running script 2 calculated model input variables such as solar time, saturated dissolved oxygen concentration, river depth, and estimated a proxy for light intensity at the river surface, and produced an output file compatible with the requirements of *streamMetabolizer*. The script 2 calculations were based on published functions^34^, except for the new method of estimating river depth discussed in the “River depth estimation” section.

**Table 4:** Summary documentation of scripts.

Script Name and Task	When and Why	Details
1_Process-Data.R Data processing & general formatting	First script in the workflow Processes raw data into input parameter time series	• Raw data from NWIS (DO, water temperature, specific conductance, discharge, and gage height) and from NOAA (air pressure) are operated on • Daily (dv) gage height is joined to gage dataframe for sites where it is the only gage data available for a site • Air pressure data from NOAA is joined and formatted • Salinity is calculated from specific conductance • Converts data to metric units when applicable • Time matches all series to 15-minute timesteps • Fills gaps that are < 3 hours by linear approximation
2_Prepare-Model-InputFiles.R Model-specific formatting & calculations	Second script in workflow Combines the processed source data into required *streamMetabolizer* model input format	• Merges DO, water temperature, salinity, discharge, gage height, and barometric pressure data into one data frame • Calculates river depth using 1) USGS field measurements, and/or 2) using published hydraulic geometric coefficients • Converts Coordinated Universal Time (UTC) to solar time • Calculates saturated concentration of dissolved oxygen and light intensity using *streamMetabolizer* functions • Exports model input files prepared for *streamMetabolizer*
3_Verify-Model-InputFiles.R *(optional)* Plot formatted data and compare with processed source data for consistency	Third script in workflow; skipped after user gains confidence Visual check confirms integrity of model input from script-2	• Reads the initially processed data for DO, temperature, and discharge (do.csv. temp.csv, disch_gage.csv) • Reads prepared model input file • Plots processed data versus input data as a consistency check for DO, temperature, and discharge that verifies integrity of model input file
4_Run-streamMetabolizer.R Run *streamMetabolizer* model	Fourth script in workflow Provides input file and runs *streamMetabolizer;* guides re-run to improve model convergence if needed	• Reads the model input file • Reads lnQ min and max needed for partial pooling • Runs *streamMetabolizer* model • Model re-runs with more burn-in steps if R^2^ of ER-K_600_ > 0.5 (high ER-K_600_ correlation) or if ${\widehat{{\rm{R}}}}_{{{\rm{\sigma }}}_{obs}}$, ${\widehat{{\rm{R}}}}_{{{\rm{\sigma }}}_{proc}}$, or ${\widehat{{\rm{R}}}}_{{{\rm{\sigma }}}_{K600}}$ >1.1 (exceeds model convergence threshold) • Export input data, modeled DO, final model daily outputs, and model_performance_summary.csv with performance diagnostic metrics
5_PostProcess-ModelOutputs.R Flag/censor modeled outputs based on criteria and create plots for analysis	Fifth script to be run in workflow Provides versions of flagged and censored model outputs using diagnostics delivered by model, creates plots for analysis	• Reads in model input and output file • Flags daily output using four criteria to help identify potentially unreliable model estimates • Exports complete model output .csv with flags as well as a censored .csv that removes output for days with any flag • Exports pdf of plots that include GPP, ER, NEP, K_600_, discharge, and depth; DO daily range, DO fraction saturation range, and temperature for analysis • Additional plots can be enabled

Running script 3 provided a consistency check with script 1 outputs before running script 4 to run the *streamMetabolizer* model. Script 5 post processes the model outputs to produce results and model diagnostics where daily metabolism results are flagged based on established criteria^34^. Also provided are plots for visual evaluation of the results as well as censored versions of metabolism output files that remove results for all days that were flagged. Details are provided in the “Quality assurance” section. Table 4 summarizes script operation in data acquisition, preparation of inputs, running the model, and post-processing outputs to evaluate and quality assure the model results.

### Running the metabolism model

We ran *streamMetabolizer* version 0.12.0 on a laptop using R version 4.1.1^37^. Computational times varied between 1 and 12 hours per site, with the two IRB sites with more than 5 years of record (Kankakee River at Davis and Illinois River at Florence) needing to be split into approximately 3-year segments to facilitate run completion. We used the *streamMetabolizer* option for Bayesian partial pooling in our models, which conditions estimates of K_600_ based on the expectation that K_600_ varies as a function of discharge. Appling *et al*.^33^ showed that partial pooling helps improve model performance because, although partial pooling does not impose a strict relationship between K_600_ and discharge, it establishes an across-day, piecewise linear relationship between ln(K_600_) and ln(*Q*) that helps improve the estimation of GPP, ER, and K_600_. Models were run with the recommended setup using four Monte Carlo Markov Chains and 1000 warmup steps. The *streamMetabolizer* model calculates values of the Gelman-Rubin statistic for observational error, ${\widehat{{\rm{R}}}}_{{{\rm{\sigma }}}_{obs}}$, process error, ${\widehat{{\rm{R}}}}_{{{\rm{\sigma }}}_{proc}}$, and K_600_ estimation error, ${\widehat{{\rm{R}}}}_{{{\rm{\sigma }}}_{K600}}$, with values ≤ 1.1 used as an initial screening criteria to indicate that model converged adequately^38,39^. Many of the IRB models converged on first run, but if unsuccessful, we ran the models again after increasing the number of burn-in steps to 1500. After the model runs were completed, we compiled the results and used the final diagnostic values reported by *streamMetabolizer* in our quality assurance steps. Also, at several river sites we tested the influence of using the default initial values for GPP, ER, and K_600_ provided in *streamMetabolizer* by varying initial values by approximately a factor of two and finding that model outcomes were robust.

### Quality assurance

Daily model outputs were flagged based on indicators of poor signal to noise strength of the modeled timeseries, and indicators of biologically and physically unrealistic outcomes for GPP, ER, and K_600_. For Flag 1, we compared each day’s coefficient of determination of modeled oxygen, R^2^_det_ against a threshold to assess signal to noise strength. For Flag 2 and 3, we assessed biologically unrealistic values of GPP and ER, respectively, following a previous example^34^ that allowed for slightly negative GPP and slightly positive ER outcomes to reflect error variation. Lastly, for Flag 4 we assessed physically unrealistic values of K_600_ (Table 5).

**Table 5:** Flagging of daily estimates of GPP, ER, and K_600_ and confidence criteria for overall metabolism outcomes at IRB river sites.

Flags and Metrics	Criteria Description	Quality Assurance Assessment
Daily flags		
Flag 1	low signal to noise ratio	flag the daily values of GPP, ER, and K_600_ when R^2^_det_ for that day < 15th percentile of the daily R^2^_det_ values and if 15th percentile of R^2^_det_ values < 0
Flag 2	biologically unrealistic GPP	flag the daily value of GPP, if GPP < −0.5
Flag 3	biologically unrealistic ER	flag the daily value of ER, if ER > + 0.5
Flag 4	unrealistically high K_600_	flag the daily value of K_600,_ if K_600 _> 20
By-site confidence metrics		
Confidence criterion 1	% of days with biologically unrealistic GPP < −0.5	**HIGH** if % days with biologically unrealistic GPP < 25%
		**MEDIUM** if % days with biologically unrealistic GPP ≥ 25% but < 50%
		**LOW** if % days with biologically unrealistic GPP ≥ 50%
Confidence criterion 2	% of days with biologically unrealistic ER > 0.5	**HIGH** if % days with biologically unrealistic ER < 25%
		**MEDIUM** if % days with biologically unrealistic ER ≥ 25% but < 50%
		**LOW** if % days with biologically unrealistic ER ≥ 50%
Confidence criterion 3	range of K_600_ values for model period unrealistically large	**HIGH** if 90^th^ – 10^th^ percentile K_600_ < 15
		**MEDIUM** if 90^th^ – 10^th^ percentile K_600_ -between 15 and 50
		**LOW** if 90^th^ – 10^th^ percentile K_600_ > 50
Confidence criterion 4	model convergence statistics ($\widehat{{\rm{R}}}$) exceed criteria	**HIGH** if both ${\widehat{{\rm{R}}}}_{{{\rm{\sigma }}}_{proc}}$, and ${\widehat{{\rm{R}}}}_{{{\rm{\sigma }}}_{K600}}$ <1.2
		**LOW** if one or both convergence statistics ≥ 1.2
Confidence criterion 5	% of days that nearest upstream flow regulation was within the “metabolism reach length”	**HIGH** if distance to upstream flow regulation > metabolism reach length for more than 80% of days
		**MEDIUM** if distance to upstream flow regulation > metabolism reach length for more than 50% but less than 80% of days
		**LOW** if distance to upstream flow regulation < metabolism reach length for more than 50% of days

Our overall confidence assessments in metabolism outcomes followed Appling *et al*.^34^ (Table 5). We assessed the percentage of days that estimated GPP, ER, and K_600_ fell outside biologically or physically realistic thresholds as well as assessing model convergence statistics ($\widehat{{\rm{R}}}$) that could indicate inadequate convergence of parameter estimates. Lastly, we assessed potential interference in metabolism estimation depending on proximity of nearest upstream dam or lake (Table 5).

To evaluate overall confidence in metabolism results for IRB rivers, we ranked each river based on combining the individual rankings for the five criteria [(Table 5)]. A river site’s individual ratings needed to be high for all five metrics for that site’s metabolism overall output to rank as “High” in confidence. A single low rating for any criterion earned a “Low” overall confidence assessment. All other combinations of individual ratings earned a “Medium” overall confidence assessment for a river site’s estimated metabolism (Table 5).

## Data Records

Our U.S. Geological Survey data release^8^ (10.5066/P9TEBOUR) presents long-term aquatic metabolism estimation at 17 river sites in the IRB. The principal outcomes are 15,176 daily estimates of GPP, ER, and K_600_ accompanied by sub-daily input timeseries of dissolved oxygen, temperature, barometric pressure, and river depth and discharge, as well as diagnostic metrics and statistics which we used to assess the quality of model outcomes. Our source data for the IRB (Table 1) had only minimal overlap encompassing a partial record for one site, DES PLAINES RIVER AT JOLIET, IL, with a previous multi-river modeling study^40^.

Metabolism estimates for the Illinois River and Fox River indicate that autotrophic conditions occur between 14 and 56% of days compared to the Kankakee and Des Plaines Rivers, which experienced autotrophy on just a few percent of days (Table 6). Metabolism in the regulated rivers of the IRB can be informative about hydrologic, biogeochemical, and ecosystem health issues in larger rivers managed for navigation. We particularly encourage use of the IRB river metabolism data^8^ by joining with other IRB data sets^24^ to identify and isolate drivers and develop early warning indicators of planktonic algal blooms in rivers.

**Table 6:** Time-averaged IRB river discharge, metabolism, and percent of days at each site with autotrophic metabolism, i.e. NEP > 0.

Site Name	NWIS Number	Number days w/o flags (%)	Mean River Discharge ± s.d. (m^3^ s^−1^)	Mean Metabolism Value ± s.d.			% days auto-trophic
				GPP	ER (g O_2_ m^−2^ d^−1^)	NEP	
**ILLINOIS RIVER** AT FLORENCE, IL	05586300	1888 (73%)	**892.1 ± **670.4	**2.22 ± **2.28	−**6.14 ± **4.37	−**3.92**	**14**
**ILLINOIS RIVER** AT HENRY, IL	05558300	444 (68%)	**532.0 ± **423.3	**2.41 ± **2.29	−**7.44 ± **4.55	−**4.98**	**22**
**ILLINOIS RIVER** AT STARVED ROCK, IL	05553700	398 (59%)	**348.4 ± **327.3	**4.87 ± **4.33	−**5.17 ± **4.82	−**0.30**	**56**
**ILLINOIS RIVER** AT SENECA, IL	05543010	337 (32%)	**353.2 ± **293.4	**4.14 ± **3.49	−**8.01 ± **6.06	−**3.87**	**39**
**FOX RIVER** NEAR MCHENRY, IL	05549500	351 (95%)	**123.6 ± **12.8	**8.64 ± **5.04	−**7.79 ± **4.22	**0.85**	**47**
**FOX RIVER** AT ALGONQUIN, IL	05550001	351 (84%)	**47.1 ± **42.1	**5.62 ± **3.01	−**6.83 ± **4.19	−**1.21**	**38**
**DES PLAINES RIVER** AT ROUTE 53 AT JOLIET, IL	05537980	767 (75%)	**135.7 ± **92.6	**3.76 ± **2.07	−**8.78 ± **2.21	−**5.02**	**5**
**DES PLAINES RIVER** AT ROCKDALE, IL	05538010	174 (42%)	**118.4 ± **67.4	**0.70 ± **1.15	−**7.57 ± **3.76	−**6.87**	**1**
**DES PLAINES RIVER** IN LOCK CHANNEL AT ROCKDALE, IL	05538020	266 (44%)	**24.9 ± **66.8	**1.09 ± **0.99	−**4.41 ± **3.39	−**3.32**	**10**
**KANKAKEE RIVER** AT SHELBY, IN	05518000	1325 (89%)	**61.2 ± **29.4	**0.45 ± **0.69	−**3.78 ± **2.28	−**3.35**	**1**
**KANKAKEE RIVER** AT DUNNS BRIDGE, IN	05517500	556 (96%)	**47.2 ± **25.7	**0.59 ± **0.51	−**4.23 ± **1.90	−**3.65**	**2**
**KANKAKEE RIVER** AT DAVIS, IN	05515500	1630 (88%)	**19.6 ± **8.2	**1.01 ± **0.85	−**5.42 ± **2.11	−**4.41**	**0**
**IROQUOIS RIVER** NEAR FORESMAN, IN	05524500	439 (83%)	**12.4 ± **13.8	**2.47 ± **3.56	−**5.79 ± **3.84	−**3.32**	**16**
**GRAND CALUMET RIVER** AT COLUMBIA AV AT HAMMOND, IN	05536356	167 (94%)	**4.2 ± **0.7	**1.64 ± **0.79	−**2.56 ± **0.96	−**0.92**	**16**
**LICK CREEK** NEAR WOODSIDE, IL	05576100	453 (67%)	**1.3 ± **2.5	**2.67 ± **3.03	−**7.84 ± **3.39	−**5.17**	**12**
**SUGAR CREEK** NEAR CHATHAM, IL	05576195	234 (28%)	**1.7 ± **6.1	**2.39 ± **2.29	−**7.46 ± **4.54	−**5.06**	**18**
**KICKAPOO CREEK** NEAR BLOOMINGTON, IL	05579630	1158 (92%)	**0.4 ± **1.8	**2.32 ± **2.35	−**3.61 ± **2.85	−**1.29**	**23**

### Data release file structure

Our data release^8^ provides files documenting metabolism estimation for 17 IRB rivers and the associated workflow. The main landing page of the USGS data release includes the metadata, readme file, and scripts (R code), and from there two child items that can be accessed leading to “Input data” and “Output data” pages, each with additional metadata and downloadable files. The data release can be accessed at 10.5066/P9TEBOUR. The structure of the data release and locations of downloadable files are summarized below:

**MAIN PAGE: Metadata File, Readme File, and Scripts**

- **RiverMET_workflow_and_scripts_metadata.xml**: Metadata file describing overview of workflow and scripts
- **RiverMET_readMe.txt**: Readme file providing overview of file contents and guidance for running the scripts
- **RiverMET_Scripts.zip**: R code scripts 1 through 5 are provided and can be downloaded with this zip file. For convenience, we list the Script names and note behind each Script the input and output files that are downloadable under Child Item 1 (Inputs) and Child Item 2 (Outputs) as described further below:
  - **1_Process-Data.R** (note: Script-1 input files not included but output from Script-1 is provided in the form of Script-2 input files)
  - **2_Prepare-Model-InputFiles.R** (note: Script 2 input files included, see Child Item 1; Script-2 output files also included and are equivalent to Script-3 and Script-4 input files, see Child Item 2)
  - **3_Verify-Model-InputFiles.R** (note: Script-3 output files not included because this is an optional step for cross checking files)
  - **4_Run-streamMetabolizer.R** (note: Script-4 output files are not included because they are not useful without first being processed by Script-5)
  - **5_PostProcess-ModelOutputs.R** (note: Script-5 output files are included, see Child Item 2)

**CHILD ITEM 1: Input Files**

- **RiverMET_Input_Files_metadata.xml**: Metadata file describing all input data including column headers and data units.
- **RiverMET_Inputs.zip:** Downloadable Script 2 input files with **filenames** and contents summarized below.

- **barop.csv** – barometric pressure in millibar (mb); 15-minute time series
- **disch_gage.csv** – discharge in m^3^ s^−1^, gage height in m; 15 – minute time series
- **do.csv** – dissolved oxygen in mg/L; 15-minute time series
- **sal.csv** – salinity in Practical Salinity Units (PSU); 15-minute time series
- **temp.csv** – water temperature in degrees Celsius (°C); 15-minute time series
- **hydraulic_coeffs.txt** – hydraulic geometry coefficients *a, b, c*, and *f* as used in estimation equations for river width, *B* = *aQ*^*b*^ and river depth, *h = cQ*^*f*^ where *Q* is river discharge, *B* is river width, and *h* is river depth.

**CHILD ITEM 2: Output Files**

- **RiverMET_Output_Files_metadata.xml:** Metadata file describing all output data including column headers and data units.
- **RiverMET_Outputs.zip**: Downloadable output files in two folders, “outputs_from_script-2” and “outputs_from_script-5”. Script-2 output files are ready for modeling using *streamMetabolizer*. Script-5 output files are the final metabolism outputs from our study. Output files details are described below:
  - **RiverMET_Outputs.zip/outputs/outputs_from script-2/**: (note: 34 csv files with 17 using hydraulic geometry estimation of river depth and 17 using gage height estimation of river depth; example filename: **bayesInput_[date]_depth-hgc_[site_no].csv**
  - **RiverMET_Outputs.zip/outputs/outputs_from_script-5/**: (note: “outputs_from_script-5” has two folders, “outputs-A” and “outputs-B”. Each folder has 21 files including 15 site files plus 3 files each for 2 long-record sites. The “outputs-A” filenames follow this example: **flagged_GPP_ER_K600_[date]_depth-hgc_[site_no].csv**. The “outputs-B” filenames follow this example: **censored_ GPP_ER_K600 _[date]_depth-hgc_[site_no].csv**.

## Technical Validation

There is no universally accepted way to quality assure modeling results. In the IRB we assessed daily metabolism results by flagging values that exceeded thresholds based on biologically or physically unrealistic values or on daily model-fit diagnostics from the *streamMetabolizer* model (Table 5). Overall confidence in each river site’s model outcomes was assessed using aggregated metrics and statistical diagnostics, e.g., percentages of daily values that were flagged and model convergence statistics (Table 5).

In the IRB an average of 29% of the modeled days had one or more flags. As described in the section on “Data release file structure”, two output versions were produced that can serve various needs. The first output version provides only censored GPP, ER, and K_600_ model estimates of the highest apparent quality after removing all days with flags. However, it is possible that some “useful” data may have been removed in the censoring process. The second output version provides complete results, including results for days with flags, which allows the user to judge each day’s data and allows users to perform custom assessments of the quality of model outcome to meet specific needs.

In terms of overall confidence in model outcomes, thirteen of the seventeen IRB river metabolism timeseries earned an overall high or medium confidence ranking (Table 7). The most frequent criterion causing a low confidence ranking was exceedance of the ${\widehat{{\rm{R}}}}_{{{\rm{\sigma }}}_{K600}}$ statistic threshold of 1.2 indicating problems with model convergence. The four river sites earning a low confidence ranking were FOX RIVER NEAR MCHENRY, IL; ILLINOIS RIVER AT FLORENCE, IL; SUGAR CREEK NEAR CHATHAM, IL; and LICK CREEK NEAR WOODSIDE, IL.

**Table 7:** Summary of metabolism model confidence assessment for the 17 river sites in IRB.

NWIS Site Name	NWIS Site Number	Preferred water depth estimation method	Discharge estimation notes	Results of Confidence Assessment
**ILLINOIS RIVER** AT FLORENCE, IL	05586300	Field measurements	No continuous or daily discharge available: daily discharge estimated based on field measurements	**Low confidence**: low rating based on ${\widehat{{\rm{R}}}}_{{{\rm{\sigma }}}_{K600}}$ threshold exceedance criterion
**ILLINOIS RIVER** AT HENRY, IL	05558300	Hydraulic geometry coefficients		**High confidence**: metrics good but potential for channel exchange with large upstream ponds noted
**ILLINOIS RIVER** AT STARVED ROCK, IL	05553700	Hydraulic geometry coefficients	Replacement discharge site used (05543500)	**Medium confidence:** medium rating for percentages of days with positive ER
**ILLINOIS RIVER** AT SENECA, IL	05543010	Field measurements from replacement site (05543500)	Replacement discharge used (05543500)	**Medium confidence:** medium ratings for percentages of days with positive ER and unrealistically high gas exchange
**FOX RIVER** NEAR MCHENRY, IL	05549500	Field measurements	No continuous or daily discharge available: daily discharge estimated based on field measurements	**Low confidence**: low rating based on ${\widehat{{\rm{R}}}}_{{{\rm{\sigma }}}_{K600}}$ threshold exceedance criterion
**FOX RIVER (TAILWATER)** AT ALGONQUIN, IL	05550001	Field measurements		**Medium confidence:** at times the upstream dam was within the metabolism reach length
**DES PLAINES RIVER** AT ROUTE 53 AT JOLIET, IL	05537980	Field measurements		**Medium confidence:** at times the upstream dam was within the metabolism reach length
**DES PLAINES RIVER** AT ROCKDALE, IL	05538010	Hydraulic geometry coefficients	Replacement discharge used (05537980)	**Medium confidence:** medium rating for % of days with positive ER
**DES PLAINES RIVER** IN LOCK CHANNEL AT ROCKDALE, IL	05538020	Hydraulic geometry coefficients	No discharge available: instead it was estimated from field measurements	**Medium confidence:** metabolism reach length often shorter than lock, but may disqualify site unless user is interested in lock water quality
**GRAND CALUMET RIVER** AT COLUMBIA AV AT HAMMOND, IN	05536356	Hydraulic geometry coefficients	No discharge available: instead it was estimated from field measurements	**High confidence in results**
**KANKAKEE RIVER** AT SHELBY, IN	05518000	Field measurements		**High confidence in results**
**KANKAKEE RIVER** AT DUNNS BRIDGE, IN	05517500	Field measurements		**High confidence in results**
**KANKAKEE RIVER** AT DAVIS, IN	05515500	Field measurements		**High confidence**
**IROQUOIS RIVER** NEAR FORESMAN, IN	05524500	Field measurements		**High confidence in results**
**LICK CREEK** NEAR WOODSIDE, IL	05576100	Field measurements		**Low confidence**: low rating based on ${\widehat{{\rm{R}}}}_{{{\rm{\sigma }}}_{K600}}$ threshold exceedance criterion
**SUGAR CREEK** NEAR CHATHAM, IL	05576195	Field measurements		**Low confidence**: low rating based on ${\widehat{{\rm{R}}}}_{{{\rm{\sigma }}}_{K600}}$ threshold exceedance criterion
**KICKAPOO CREEK** NEAR BLOOMINGTON, IL	05579630	Field measurements		**High confidence**

The confidence assessment was based in a combined evaluation of 5 criteria described in Table 5.

Having approximately three quarters of the IRB river sites (76%) earn a high or medium confidence ranking is only slightly lower performance than a similarly assessed set of rivers modeled by Appling *et al*.^ 34^, where 84% ranked high or medium confidence. The IRB river metabolism results^8^ are therefore quality assured based on application of the best available diagnostic metrics and statistical criteria for models of this type. Nonetheless, it is important to consider that model confidence assessments are only guidance and do not override future investigations of model quality that may be more detailed or judged “fit for purpose”.

## Usage Notes

Our data release^8^ provides metabolism outcomes and documents our workflow for modeling metabolism at 17 ILB river sites. Here we summarize descriptive information about the dataset and guidance for its use, including geographic coordinates and period of data availability for each site (Table 1), summary of USGS parameter codes used for downloading (Table 2), information about calculating parameters needed as model inputs (Table 3), an overview of script workflows (Table 4), quality assurance criteria (Table 5), and metabolism outcomes (Table 6) including a model performance assessment (Table 7). In addition, our data release^8^ provides guidance for potential reuse of codes in the file *RiverMET_readMe.txt*, including suggestions for changes that may be needed to run on a different system, re-run IRB sites with different options, or adapt scripts to model metabolism in other rivers. Users who wish to adapt parts of our workflow will need to acquire publicly available data from USGS and NOAA. They can use existing software (dataRetrieval^29^) to download the needed USGS data from their sites of interest, including dissolved oxygen, water temperature, specific conductance, discharge, gage height, and field measurements of channel parameters from the USGS NWIS site, and they can obtain barometric pressure data from NOAA. After downloading their own data, users can adapt parts of 1_Process-Data.R to perform the data time matching, gap filling, and unit conversion (Table 4). As long as their code produces output files that match the input files for 2_Prepare-Model-InputFiles.R that we provide in our data release, they can likely make minor adaptations to run scripts 2, 3, 4 and 5 (as described in Table 4) to prepare final model inputs, run streamMetabolizer, and organize and quality assure their metabolism modeling results.

Our data release^8^ also suggests approaches that can help expand the capacity for modeling river metabolism. For example, several of the IRB sites could perhaps have been included in an earlier study^40 ^, however, not all the needed input data were available at certain sites, resulting in those sites being passed over. To facilitate modeling at those sites, where appropriate, we acquired the missing measurements from nearby “replacement” sites (Table 7). An example is several sites where dissolved oxygen was collected without collecting the river discharge needed to accomplish Bayesian partial pooling that estimates K_600_ based on a prior expectation that K_600_ varies as a function of discharge. In such cases we “replaced” the missing discharge with data from a nearby site, which allowed metabolism estimation at sites previously overlooked because of missing data^8^. Because of the large river size where replacement discharges were used, e.g., often over 350 m^3^ s^−1^, and given the proximity of the replacement site, usually within 10-km, we did not perform scaling by basin size when applying a replacement discharge.

## Data Availability

Our workflow includes scripts that were written and tested using R version 4.1.1. The scripts can be accessed from the data product^8^ which includes an appropriate licence (CC0 1.0 Universal) license permitting reuse without restrictions.
